# The therapeutic potential of angiotensin-converting enzyme inhibitor enalapril to ameliorate muscle atrophy in a murine model

**DOI:** 10.17179/excli2023-6822

**Published:** 2024-04-25

**Authors:** Sima Seifi, Seyedeh Elnaz Nazari, Amir Avan, Nima Khalili-Tanha, Fereshteh Asgharzadeh, Fatemeh Babaei, Ghazaleh Khalili-Tanha, Seyyedeh Zahra Asghari, Mahdieh Darroudi, Gordon A. Ferns, Abdoljalal Marjani, Majid Khazaei

**Affiliations:** 1 Metabolic Disorders Research Center, Department of Biochemistry and Biophysics, Golestan University of Medical Sciences Gorgan, Golestan Province, Iran; 2 Metabolic Syndrome Research Center, Mashhad University of Medical Sciences, Mashhad, Iran; 3 Department of Medical Physiology, Faculty of Medicine, Mashhad University of Medical Sciences, Mashhad, Iran; 4 Basic Sciences Research Institute, Mashhad University of Medical Sciences, Mashhad, Iran; 5 Medical Genetics Research Center, Mashhad University of Medical Sciences, Mashhad, Iran; 6 Brighton & Sussex Medical School, Department of Medical Education, Falmer, Brighton, Sussex BN1 9PH, UK

**Keywords:** muscle atrophy, limb immobilization, oxidative stress, enalapril, inflammation, renin-angiotensin system

## Abstract

Muscle atrophy due to limb immobilization and inactivity is a common consequence of many diseases and treatment processes. One of the systems activated in inflammatory conditions is the renin-angiotensin system (RAS). The present study was conducted with the aim of investigating the effects of one of the angiotensin-converting enzyme (ACE) inhibitors, enalapril, on improving muscle atrophy caused by immobility. The study was conducted in three groups: a control, an atrophy, and an atrophy group treated with enalapril on Balb/c mice. After tying a splint to cause atrophy in one of the legs, daily treatment with enalapril intraperitoneally (dissolved in DMSO) at a dose of 10 mg/kg/day was done for 7 days. On the eighth day, the splint was opened and half of the mice were evaluated. Then, in the recovery phase, treatment with enalapril was continued in the remaining mice for 10 days without a splint. At the end of each phase, the mice were examined for the muscle strength of the lower limb muscles, and histological and biochemical analyses were subsequently carried out. The tissue level of the oxidative stress index MDA was evaluated, which showed a significantly lower level in the enalapril group compared to the atrophy group (*P<0.1). Also, inflammatory factors in the enalapril group showed a decrease compared to the atrophy group. The strength of four limbs in the mice of the treatment group (-18.36 ± 1.70 %) was significantly higher than that of the atrophy group (-30.33 ± 3 %) at the end of the atrophy phase and also after 10 days of recovery. The results suggest that the use of enalapril that reduces the activation of angiotensin II-dependent pro-oxidant and pro-inflammatory pathways may improve the functional disorder and muscle necrosis in the murine model of muscle atrophy.

## Introduction

Muscular atrophy is the loss of skeletal muscle mass, which may be caused by inactivity, aging, malnutrition, medications, or a wide range of diseases or injuries that affect the nervous or musculoskeletal system (Cohen et al., 2015[5]; Rommersbach et al., 2020[16]) . Loss of skeletal muscle mass comes with poor quality of life, increased risk of morbidity, high health care costs, and increased mortality. The majority of orthopedic injuries and non-orthopedic diseases require respectively limb immobilization and bed rest and during the first 2 to 3 weeks of this period, atrophy occurs with a loss of about 0.5 % of total muscle mass per day (Wall et al., 2014[18]). Continued loss of muscle mass reduces muscle strength and impairs movement. Considering that muscle atrophy due to immobility is not fully and quickly reversed with light sports exercises, it is important to explore a method to prevent or reduce the amount of atrophy during immobility and inactivity can help maintain mass muscle and as the person's physical performance. It has been found that immobility and inactivity are associated with increased inflammation. The expression of inflammatory cytokines can be related to the progression of atrophy. Immobility-induced inflammation can activate an atrophic program by activating the transcription factor NF-κB (Van't Klooster et al., 2020[17]). During chronic inflammation, one of the mechanisms that cause muscle atrophy is increased oxidative stress (OS) (Powers, 2014[14]). NF-κB activation associated with increased ROS production plays a vital role in increasing the expression of pro-inflammatory cytokines such as TNF-α. Then, pro-inflammatory cytokines may stimulate the migration and infiltration of inflammatory cells and the secondary wave of ROS production, further amplifying the inflammatory cascade and damage (Xin et al., 2016[20]). One of the strategies to reduce the occurrence and prevention of this problem includes reducing inflammatory reactions and reducing the production of oxidative stress.

The renin-angiotensin system (RAS) is one of the systems that is activated in inflammatory conditions. Angiotensin-converting enzyme (ACE) is a critical component of the renin-angiotensin system (RAS) that is involved in the formation of angiotensin II. Ang-II produced by the ACE enzyme is claimed to exert pro-inflammatory and pro-oxidant effects. Both the proinflammatory and stressor roles of angiotensin II are mediated through stimulation of the angiotensin II type 1 receptor (AT1R) (Nataraj et al., 1999[13]). Ang-II increases NADPH-oxidase activity through receptor-dependent pathways, leading to overproduction of superoxide anion, the major reactive oxygen species (ROS) (Wei et al., 2008[19]). Activation of ROS-dependent signaling pathways induced by Ang-II may maintain inflammation and increase NF-κB activation and pro-inflammatory cytokine production (Arthur et al., 2008[1]).

Repurposing drugs that are already approved for human use, is an attractive approach for introducing new therapies. For example, there is some evidence that angiotensin-converting enzyme (ACE) inhibitors, commonly used as antihypertensive drugs, even in people who do not have cardiovascular disease, it has various beneficial effects, including reducing inflammation, and generally have few side effects (Benigni et al., 2010[2]; Chang and Wei, 2015[4]). Enalapril is an ethyl ester prodrug, its therapeutic effects are mediated by its active metabolite, enalaprilat (MK422). Enalapril is an angiotensin-converting enzyme (ACE) inhibitor and is widely used in therapeutic interventions for hypertension. Some studies have shown that the therapeutic benefits of the antihypertensive drug enalapril may be partially due to its antioxidant properties, which inhibit the production of free radicals (Brunner et al., 1981[3]). Considering that musculoskeletal abnormalities exist in various conditions, systemic factors such as the renin-angiotensin system may be a common pathway in their development. The current study evaluated the potential impact of inhibition of the RAS system in reducing inflammatory and oxidant reactions in improving muscle atrophy. To this aim, the effect of enalapril, an ACE inhibitor, was investigated in preventing and improving muscle atrophy due to immobility in the soleus and gastrocnemius muscles of the leg in Balb/c mice in a 17-day period in two phases of atrophy and recovery.

## Materials and Methods

### Enalapril

Enalapril was purchased from Medkoo Inc (catalog number 318676) and was used at a dose of 500 mg, and was dissolved in DMSO for injection according to the manufacturer's instructions.

### Animals

The ethical committee of Mashhad University of Medical Sciences approved the animal experimental protocols. The experiment was conducted on male BALB/c mice (aged 7-8 weeks old and weighing approximately 25 g), which were prepared in the Center for Reproduction and Breeding of Laboratory Animals in the School of Medicine of Mashhad University of Medical Sciences, Iran. Thirty-six mice were randomly assigned into three groups, which are listed below: control or healthy group (n = 12), Immobility group (n = 12), and Enalapril-treated group (n=12). The living conditions were standard, including 12 hours of light and dark cycles during the experiment with unlimited and free access to water and food.

### Model induction

**Figure 1:**
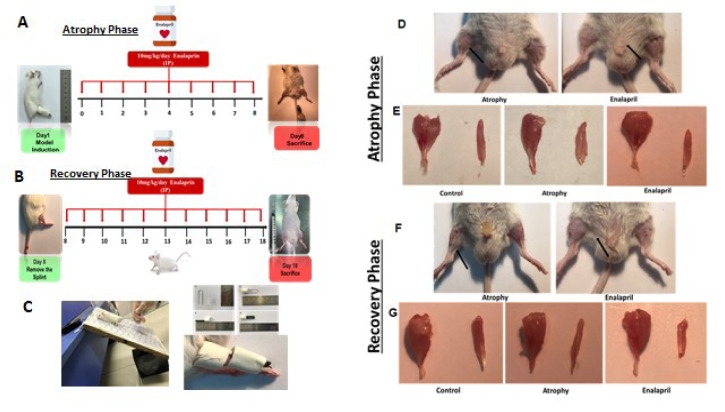
Study design and muscles macroscopic picture of model system in two phases of atrophy and recovery. A-C) The treatment period was 18 days, including 7 days of immobility in one limb (A), then on the eighth day, the mice's splints were opened and 10 days for recovery (B). Tools for modeling immobility and atrophy (C). Image of a mouse subjected to unilateral hindlimb immobilization (D). D-F) Atrophy is characterized by the loss of muscle tissue, the leg muscle of mice atrophied in two groups of atrophy and enalapril in two stages of atrophy and recovery is indicated by a black arrow, respectively. E and G) The picture of the gastrocnemius and soleus muscles in the three studied groups at the end of the atrophy and recovery phase, respectively (E), (G)

To create a model of immobility and atrophy, the method used in previous studies is applied. In short, with very simple and accessible tools, a splint is prepared for the animal, and the animal's right leg is immobilized in a position where the knee is in the extension position and the ankle is in the plantar flexion position (Figure 1(Fig. 1)) (You et al., 2015[21]). On day 0, all mice were weighed, and limb strength was measured using a force meter which will be thoroughly explained.

### Treatment

This study was performed in two phases atrophy and recovery (Figure 1(Fig. 1)). The treatment period was 18 days, including 7 days immobilized in one limb, then on the 8th day, the mice splints were opened, and 10 days for recovery followed. The treatment group was administered enalapril intraperitoneally at a dose of 10 mg/kg dissolved in 0.1 % (v/v) DMSO (100 µL/kg body weight) (De Cavanagh et al., 2003[7]).

### Four-limb strength test

**Figure 2:**
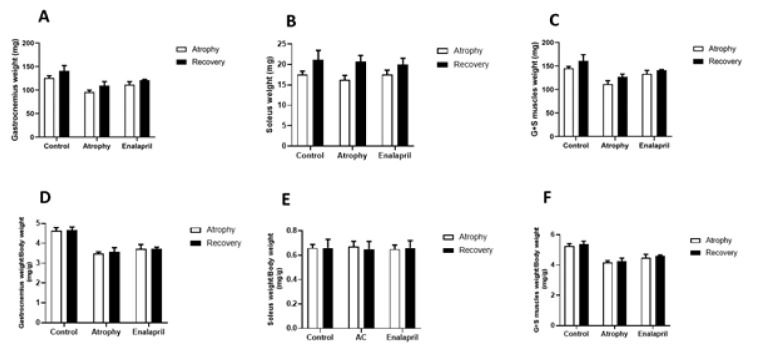
Muscles weight in the recovery phase and the atrophy phase. The mean weight of gastrocnemius muscle of mice from all three groups of control, atrophy, and treatment with enalapril was compared (A). The mean weight of soleus muscle was compared in the three groups (B). The mean weight of total of both muscles from all 3 groups were compared with each other (C). The weight of gastrocnemius muscle for each mouse was normalized to the total body weight of the mouse, which showed a significant increase in the muscle mass of the enalapril -treated group compared to the muscle weight of the atrophic group (** P < 0.01) (D). The weight of the soleus muscle for each mouse was normalized to the total body weight of the mouse, which observed no significant difference between the groups (E). The total weight of both muscles was normalized to the total body weight of mice, which showed a significant difference from the comparison of the enalapril-treated group with the atrophic group (** P < 0.01) (F)

On the 8th day, half of each group was tested for four- limb strength. Therefore, we used a thin wire mesh connected to a digital force meter (DS2-110 500 N, Toyohashi, Japan) (Figure 2(Fig. 2)). The mice were held by their tails and gently moved on the mesh; as the mice gripped it by their paws, the force was transmitted to the force meter and this measurement was repeated 2 more times for each mouse. Then the remaining mice from each group were recovered without splints for 10 days, and in the treatment group, the treatment with enalapril was continued, and mice four-limb strength was measured afterwards.

### Weighing of gastrocnemius and soleus muscles

To examine the leg muscle tissue for atrophy, the mice were sacrificed and the gastrocnemius and soleus muscle tissue was removed and examined macroscopically for atrophy (photos of these muscles were taken), and then immediately were weighted using the 0.001 g accurate electronic digital weighing scale (SF-400 10 Kg Beijing, China). This procedure was done fast so tissue water is not wasted and errors do not occur. The muscle tissue is weighted both separately and together. The target muscle tissues of each mouse were normalized with mouse weight.

### Histological studies

For the histological analysis, a part of the muscle tissue was placed in a 4 % formaldehyde-neutralized buffer and embedded in paraffin. Then the tissue sections were prepared at a thickness of 4 µm using a cryotome (Shandon, Pittsburgh, PA 15275, California USA). Light microscopy was used to examine the sections stained with Hematoxylin-Eosin (H&E) and Masson's trichrome in terms of muscle fiber size and cross-sectional area. Muscle cross-section and fiber size were analyzed using NIH Image (Image J) software.

### Biochemical analysis

A part of the muscle tissue was stored at -70 °C. This was examined after homogenization to evaluate the factors involved in muscle atrophy, including inflammatory cytokine, IL-6 level, and nitrite (NO_2-_). To prepare the muscle tissue for this test, those were homogenized and then centrifuged at room temperature and at 10000 rpm for 10 minutes. Also, blood samples were taken to measure troponin I level to evaluate muscle damage. All these factors were assessed using mouse-specific ELISA according to the manufacturer's kit (Promega Corp, Wisconsin, USA) (ZellBio GmbH, Ulm, Germany).

### Malondialdehyde (MDA) levels

PBS (phosphate buffer solution with pH 7.4) was used to homogenize the tissues. The homogenates were centrifuged for ten minutes and the supernatants were evaluated for malondialdehyde (MDA; an oxidative marker). 15 g trichloroacetic acid (TCA) was mixed with 0.375 g TBA and 2 ml concentrated hydrogen chloride and made up to a final volume of 100 ml with distilled water. Then 2 ml of the resulting solution was mixed with 1 ml of homogeneous tissue and placed in a boiling water bath for 50 minutes. After cooling the mixture, 25 μl of concentrated HCl solution was added to the tube and centrifuged at 1000 g for 10 minutes. After discarding the supernatant, the optical density (OD) of the mixture was read against blank at 535 nm. Tissue MDA content was calculated by the following equation (where C (M): concentration in molar, A: optical density). C (M) = A / 1.56 * 105

### Molecular studies

Real time PCR was used to evaluate the effect of enalapril on the mRNA expression level of inflammatory cytokines, IL-6 and TNF-α, in atrophied muscle tissue. Total RNA extraction was performed using total RNA extracted kit (Yekta Tajhiz Azma, Cat number: YT9080) according to the manufacturer's instructions. CDNA synthesis kit (TAKARA, Cat number: RR037A) was used for cDNA synthesis. The gene expression levels were measured using SYBR Ampliqon Green Moster Mix kit by ABI applied bio system. The thermal program consisted of 1 cycle of 95 °C for 15 minutes, followed by 30-40 cycles including 95 °C for 30 seconds and 60-55 °C for 60 seconds. A comparative method was used to analyze the mRNA level normalized against GAPDH (2 -∆∆Ct).

### Statistical analysis

Data were presented as mean ± standard deviation (SD) and analyzed by the one-way analysis of variance (ANOVA). It should be noted that the mice used in our experiment were in the homogenous body weight range, but to reduce even the slightest differences that could affect our evaluation, we normalized some of our results by dividing the outcomes by the body weights (***P < 0.001) (**P < 0.01) (*P < 0.05). In order to compare groups, we use a post hoc LSD test.

## Results

### Macroscopic observation

The treatment period was 18 days, including 7 days of immobility in one limb in the atrophy phase, and 10 days for recovery (Figure 1(Fig. 1)). At the end of the atrophy phase, the foot splint was opened from the mice and the closed muscle was examined macroscopically and pictures were taken. In the group treated with enalapril, the atrophied muscle had a smaller size reduction compared to the atrophied muscle in the atrophy group (Figure 2(Fig. 2)). Half of the mice in each group that entered the recovery phase had images taken with a camera of the muscle of interest after 10 days of recovery, and the images showed more muscle atrophy in the atrophy group than in the treatment group (Figure 1(Fig. 1)).

### Muscle weight

**Figure 3:**
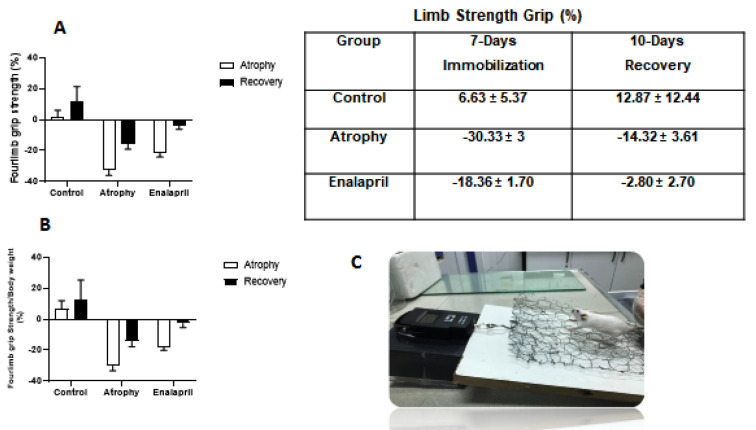
Four-limb grip strength. The limb strength of the mice atrophic group and the mice treated with enalapril group were calculated as a percentage compared to the limb strength of the control group in the stages of atrophy and recovery (A). Average limb strength normalized to the weight of each mouse in all 3 study groups and each group in two phases (B). Image of a thin wire mesh attached to a digital force gauge (DS2-110 500 N, Toyohashi, Japan) for measuring muscle strength in mice (C).

Our results showed that enalapril reduced immobility-induced muscle atrophy in the enalapril-treated group compared to the atrophy group. Atrophy phase mice from all three groups were sacrificed on the 8th day, and the gastrocnemius and soleus muscles of the right leg of the mice were removed. The weight of each muscle was checked separately and the total weight of both muscles for each mouse. Also, muscle weight was normalized with the total body weight of each mouse. The results of muscle weight were analyzed in 6 manners, including 1: Comparison of the average weight of the gastrocnemius muscle in 3 groups, in both phases of the study (Figure 2(Fig. 2)), 2: Comparison of the average weight of the soleus muscle in 3 groups in both phases of the study (Figure 2(Fig. 2)), 3: Comparison of the total weight of the two muscles in the 3 groups in both phases of the study (Figure 2(Fig. 2)), 4: Comparison of the average weight of the gastrocnemius muscle, which was normalized with the weight of the mice, in the 3 groups in both phases of the study (Figure 2(Fig. 2)), 5: Comparison of the average weight of the soleus muscle which is normalized with the weight of the mice in 3 groups in both phases of the study (Figure 3(Fig. 3)) and finally 6: the comparison of the average weight of the two muscles which is normalized with the weight of the mice in the 3 groups in both phases of the study (Figure 3(Fig. 3)). The results showed a decrease in muscle atrophy in the treatment group compared to the atrophy group, but none of the results were significant.

### Four-limb grip strength

**Figure 4:**
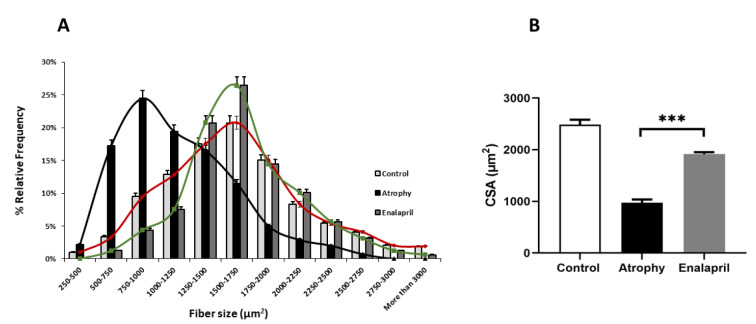
Cross-sectional area (CSA) (2 μm) of fibers in response to atrophy and enalapril treatment. A-B) Relative abundance of fiber size in square micrometers. The size of fibers increased more in the enalapril-treated group compared to the atrophy group (*** P < 0.001).

The evaluation of muscle strength was done to investigate the effect of immobility period and treatment with enalapril drug on the function of the target muscle. For this purpose, the grip strength of each mouse in the control group and the atrophy and treatment group was calculated as a percentage, where the average muscle strength of the mice on the zero-day of the study is considered to be zero percent (Figure 3(Fig. 3)). Then the limb strength of each mouse was normalized with its weight and the normalized limb strength was calculated as a percentage in all 3 groups (Figure 3(Fig. 3)). The results of the examination of muscle strength at the end of the atrophy phase showed that the strength of the enalapril-treated group (-18.36 ± 1.70 %) had a smaller decrease compared to the strength of the atrophy group (-30.33 ± 3 %) compared to the control group (6.63 ± 5.37 %). This result was also seen at the end of the recovery phase. We also evaluated cross-sectional area (CSA) (2 μm) of fibers in response to atrophy and enalapril treatment (Figure 4(Fig. 4)). 

### Histological findings

**Figure 5:**
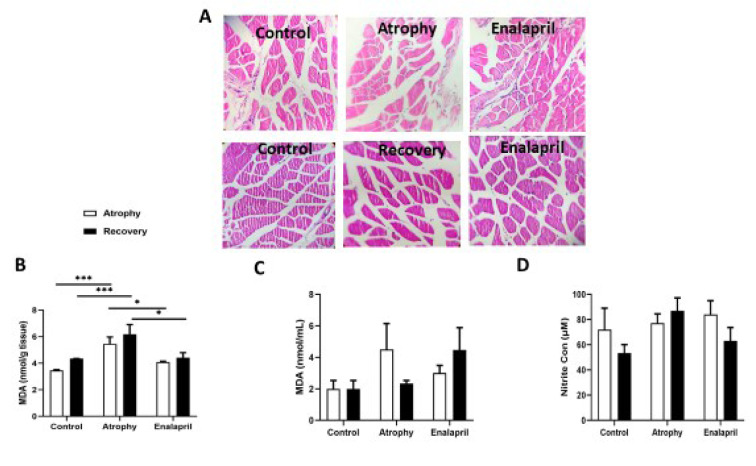
Histological examination. Image of muscle cross-section using NIH Image software (Image J) in the atrophy and recovery phases, respectively (A). B) MDA level, an oxidant index, in muscle tissue (C) and in serum samples of three study groups in two phases of atrophy and recovery. Nitrite concentration between three groups in both phases (D). Statistical significance is represented as follows: * p<0.01, ** p<0.01 and *** p<0.001.

The size of muscle fibers decreased during immobilization, and when examining the cross-section of the muscle, the number of muscle bundles in the microscopic field of view was rarer (Figure 5(Fig. 5)). The relative frequency showed that in the samples of the atrophy group, most of the fiber sizes were between 500 to 1250 μm square, and most of them corresponded to 790 to 1000 μm square. In the group treated with enalapril, the fiber size distribution changed to larger sizes, and the largest sizes are between 1250 to 1750 square meters, which indicates the positive effect of this drug in reducing atrophy as a result of immobility. Also, the cross-sectional area in the group treated with enalapril was significantly improved compared to the atrophy group (*** P < 0.001) (Figure 5(Fig. 5)).

### Biochemical and molecular tests

The level of MDA, as an oxidant index, increased after immobilization both in muscle tissues and in serum samples (Figure 5(Fig. 5)). The results showed that enalapril drug in the treatment group in the atrophy stage significantly reduced MDA levels compared to the atrophy group (*P < 0. 1). In the recovery phase, MDA assessment showed that continued treatment with enalapril significantly reduced oxidative stress levels compared to the atrophy group (* P < 0. 1).

**Figure 6:**
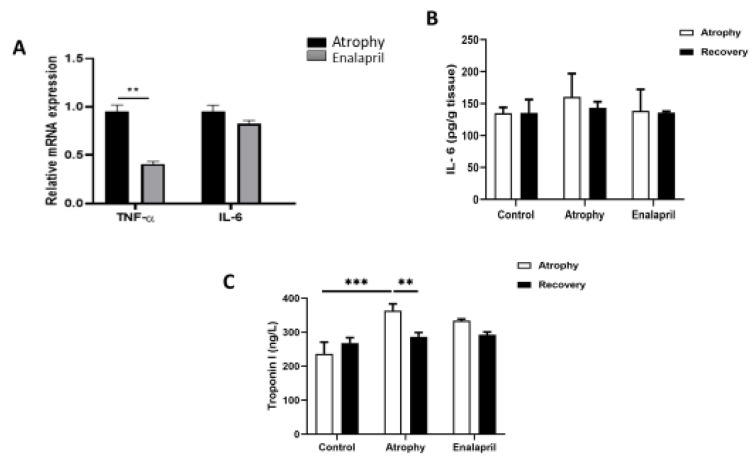
The expression level of the inflammatory cytokine. A) Comparison of the expression levels of inflammatory cytokines IL-6 and TNF-α in two atrophy and enalapril groups at the mRNA level in the target muscle tissue in the atrophy phase. B) IL-6 at the protein level in the target muscle tissue in the 3 studied groups in the two phases of atrophy and recovery. C) Troponin I (TnI) serum level, index of muscle damage, in three study groups in two phases of atrophy and recovery. Statistical significance is represented as follows: * p<0.01, ** p<0.01 and *** p<0.001.

The results of IL-6 inflammatory cytokine expression at the protein level and at the mRNA level in the target muscle tissue in the 3 study groups are shown in Figure 6(Fig. 6), respectively. The results showed that IL-6 protein expression decreased in the group treated with enalapril compared to the atrophy group, but this decrease was not significant. Also, in the results of IL-6 expression at the mRNA level, no significant difference was seen between the treatment and atrophy groups (Figure 6(Fig. 6)).

The results of the expression of the inflammatory cytokine TNF-α at the mRNA level are shown in Figure 6B(Fig. 6). The results showed that the expression of the inflammatory factor TNF-α in the group treated with enalapril significantly decreased compared to the atrophy group (**P < 0.01). These results suggest that enalapril can have protective effects against atrophy by reducing inflammatory responses as a result of immobility in the mouse model. Moreover, the serum level of troponin I in the atrophy group increased significantly compared to the control group (***P<0.001). This factor was reduced in the group treated with enalapril compared to the atrophy group, but this reduction was insignificant. There was no significant difference in troponin I serum levels in the three studied groups during the recovery phase (Figure 6C(Fig. 6)).

See also the supplementary data.

## Discussion

Muscle atrophy caused by lack of movement is due to both decreased protein synthesis and increased protein breakdown. Although several factors play a role in regulating the rate of protein synthesis and breakdown in skeletal muscle, immobility, and inactivity are associated with increased inflammation in the same organ and systemic inflammation (Van't Klooster et al., 2020[17]). Persistent expression of inflammatory cytokines is detrimental to muscle mass, as it activates signaling pathways that cause protein degradation and suppression of protein synthesis, resulting in muscle cell atrophy (Reid and Moylan, 2011[15]). A study by Haddad et al. on desert mice showed that local administration of IL-6 led to atrophy in healthy animals (Haddad et al., 2005[9]).

Ang II, one of the factors causing cardiovascular damage in hypertension, induces many pathophysiological actions by inducing ROS production through activating vascular NADPH oxidase. Ang II stimulation can increase in association with the activation of immune response and inflammation. As a result, other inflammatory events occur, such as increased production of ROS and levels of cytokines and chemokines (Dinh et al., 2014[8]). Ang-II and the resulting activation of ROS signaling may maintain inflammation, and increase NF-κB activation and proinflammatory cytokine production (Arthur et al., 2008[1]). During inflammatory processes, excessive local concentration of Ang II increases vascular permeability by stimulating inflammatory responses and production of prostaglandins and vascular endothelial growth factor (VEGF). Ang II signaling through AT1R leads to the activation of NF-κB, resulting in the production of chemokines, proinflammatory cytokines, and cell adhesion molecules by resident cells, which enhance the migration of inflammatory cells to sites of tissue damage, thereby enhancing inflammatory responses (Chang and Wei, 2015[4]). Considering that musculoskeletal abnormalities exist in variety of conditions, systemic factors such as the renin-angiotensin system may be involved as a common pathway in their development.

Kadoguchi et al. in 2015 investigated whether Ang II can directly induce musculoskeletal abnormalities. In this study, angiotensin II (1000 ng/kg/min^-1^) was administered to male C57BL/6J mice (10-12 weeks old) through subcutaneously implanted osmotic minipumps for 4 weeks. Angiotensin II significantly reduced body and hind limb skeletal muscle weights. In parallel, the cross-sectional area of the muscle was also reduced in the skeletal muscle. The production of superoxide derived from NAD(P)H oxidase increased. Working and running distances evaluated by treadmill test were significantly reduced in mice treated with Ang II (for 4 weeks) (Kadoguchi et al., 2015[10]). Therefore, it seems possible that inhibiting the RAS system may be effective in improving muscle atrophy by reducing inflammatory and oxidant reactions.

Marzetti et al. in 2013 studied the protective mechanisms of enalapril on the muscles of elderly people. TNF-α acts primarily by activating the transcription factor NF-κB, which is responsible for regulating a wide variety of genes, including iNOS. Excessive production of NO by iNOS can lead to the formation of highly toxic peroxynitrite (ONOO-) through the reaction of NO with superoxide anions (O2• -). In this study, the expression level of tumor necrosis factor-alpha (TNF-α) was examined to determine the effect of enalapril on inflammation. This study suggested that the muscle-protective effect of enalapril administered late in life may be mediated in part by reducing oxidative stress and reducing the expression of pro-inflammatory factors. Decreased expression of iNOS and TNF-α was observed in mice treated with enalapril (Marzetti et al., 2013[12]).

Angiotensin-converting enzyme (ACE) inhibitors are used clinically to control cardiomyopathy in patients with Duchenne muscular dystrophy. Angiotensin II is known to exert proinflammatory and prooxidative effects that may contribute to the early events of dystrophic muscle degeneration. Cozzoli et al. in 2011 aiming to evaluate the effects of early enalapril treatment on the pathological symptoms of the mdx mouse model (a mouse model studied in Duchenne muscular dystrophy), observed that enalapril increased forelimb strength. Decreased production of superoxide anion was observed by dihydroethidium staining in the tibialis anterior muscle, as well as a significant decrease in the activated form of proinflammatory nuclear factor-κB in rats treated with enalapril. Finally, the results suggest the ability of enalapril to reduce the activation (angiotensin II-dependent) of proinflammatory and prooxidant pathways, pathways that may partially contribute to muscle dysfunction and necrosis (Cozzoli et al., 2011[6]).

In 2023, Khan et al. conducted a study investigating the potential of enalapril as an anti-arthritic agent. In this research, three doses of enalapril were used and several pro-inflammatory and anti-inflammatory mediators and some oxidative stress parameters were targeted. The research results showed the anti-arthritic activity of enalapril in the arthritis model caused by CFA. Enalapril had a suppressive effect on paw inflammation and arthritis score. Body weight and radiological changes improved with enalapril. In addition, it normalized biochemical and hematological markers. Enalapril also showed its antioxidant potential by increasing SOD, CAT, and GSH levels while suppressing MDA levels (Khan et al., 2023[11]).

In line with these results, our results showed that the tissue level of MDA in immobilized muscle was increased in the atrophy group compared to the control group, and there may be a correlation between atrophy and MDA levels. However, intraperitoneal administration of enalapril can decrease the tissue level of MDA. Also, based on the results of reducing the expression of pro-inflammatory cytokines in the enalapril treatment group compared to the atrophy group, we hypothesized that enalapril can act as an antioxidant and anti-inflammatory agent that can improve muscles. Another finding was the quadruped grip strength test, which was clear that during the period of immobility and non-use of the limb, the target limb is affected and loses its natural strength, resulting in less force. The results of this study showed that enalapril administration increased grip strength, although this increase was not as strong as the control group. This effect was observed not only during immobilization but also during the recovery phase. Also, in histological studies on the size of fibers and the cross-sectional area of muscle tissue, it was found that the treatment with enalapril significantly increased the fiber size and cross-sectional view in terms of the number of muscle fibers and thus reduced muscle atrophy caused by immobility.

## Conclusions

Muscle atrophy is defined as a loss of the tissue. Muscle atrophy due to immobility is a common complication of many diseases (e.g., strokes and chronic diseases) and treatment processes (e.g., immobilization of limbs in orthopedic surgery and tendon repair). Considering that the loss of muscle mass is involved in the creation or exacerbation of many health problems, it is very important to investigate a method to prevent or reduce the amount of atrophy during inactivity and immobility that can help maintain muscle mass and physical performance. Various molecular factors and stimuli including oxidative stress and inflammatory cytokines (TNF-α, IL-1β, IL-6) are involved in skeletal muscle atrophy as a result of immobility, which makes these molecular factors significant therapeutic targets. One of the strategies to reduce the occurrence and prevention of this problem includes reducing inflammatory reactions and reducing the production of oxidative stress. The renin-angiotensin system (RAS) is one of the systems that is activated in inflammatory conditions. Angiotensin-converting enzyme (ACE) is a critical component of the renin-angiotensin system (RAS) that is involved in the formation of angiotensin II. Ang-II produced by the ACE enzyme is claimed to exert pro-inflammatory and pro-oxidant effects. The current study evaluated the potential impact of inhibition of the RAS system in reducing inflammatory and oxidant reactions in improving muscle atrophy. To this aim, the effect of enalapril, an ACE inhibitor, was investigated in preventing and improving muscle atrophy due to immobility. This study shows that the use of enalapril, which reduces the activation of pro-oxidant and pro-inflammatory pathways dependent on angiotensin II, can be a promising result in reducing atrophy of sedentary muscles.

## Notes

Sima Seifi, Seyedeh Elnaz Nazari and Amir Avan contributed equally as first author.

Abdoljalal Marjani and Majid Khazaei (Basic Sciences Research Institute, Mashhad University of Medical Sciences, Mashhad, Iran; Metabolic Syndrome Research Center, Mashhad University of Medical Sciences, Mashhad, Iran; Tel: +98 513 8002298, E-mail: Khazaeim@mums.ac.ir) contributed equally as corresponding author.

## Declaration

### Author contributions

SS, SEN and AA performed the experiment and drafted the manuscript; NKT, FA and FB processed the experimental data and performed the analysis; GKT, SZA and MD aided in interpreting the results and worked on the manuscript; AM and MK were involved in planning and supervising the work. All authors discussed the results and commented on the manuscript.

### Funding

This research was supported by Golestan University of Medical Sciences, grant no. 1401014 (Abdoljalal Marjani) and Mashhad University of Medical Sciences, grant no. 981618 (Amir Avan).

### Conflict of interest

The authors have no conflicts of interest to declare.

## Supplementary Material

Supplementary data
